# Efficacy and safety of teriparatide in kidney transplant recipients with osteoporosis and low bone turnover: a real-world experience

**DOI:** 10.1007/s11255-025-04383-8

**Published:** 2025-01-27

**Authors:** Daniele Vetrano, Francesco Aguanno, Alessia Passaseo, Simona Barbuto, Francesco Tondolo, Veronica Catalano, Guido Zavatta, Uberto Pagotto, Gaetano La Manna, Giuseppe Cianciolo

**Affiliations:** 1 Department of Medical and Surgical Sciences (DIMEC), Alma Mater Studiorum University of Bologna, Bologna, Italy; 2 Nephrology, Dialysis and Kidney Transplant Unit, IRCCS Azienda Ospedaliero-Universitaria di Bologna, Bologna, Italy; 3 Division of Endocrinology and Diabetes Prevention and Care, IRCCS Azienda Ospedaliero-Universitaria di Bologna, Bologna, Italy

**Keywords:** CKD-MBD, Kidney transplant, Bone turnover markers, Teriparatide, Osteoporosis

## Abstract

**Introduction:**

Kidney transplantation is the preferred treatment for end-stage kidney disease (ESKD), enhancing survival and quality of life. However, kidney transplant recipients (KTRs) are at high risk for bone disorders, particularly low bone turnover disease, which increases fracture risk. Teriparatide, an anabolic agent, may provide a beneficial treatment option for these patients.

**Materials and methods:**

This single-center, retrospective observational study involved 18 KTRs with osteoporosis, low bone turnover, and a history of vertebral or non-vertebral fractures. Patients received teriparatide (20 μg/day) for up to 2 years. Areal bone mineral density (aBMD) at the lumbar spine (LS), total hip (TH), femoral neck (FN), and trabecular bone score (TBS) were measured at baseline, 1 year, and 2 years. In addition, bone turnover markers (BTMs), serum calcium, phosphorus, parathyroid hormone (PTH), and kidney function were monitored.

**Results:**

Significant increases in LS aBMD were observed after 1 year (0.941 ± 0.152 vs 1.043 ± 0.165, *p* = 0.04) and maintained after 2 years compared to baseline (0.941 ± 0.152 vs 1.074 ± 0.154, *p* = 0.03). TH aBMD significantly increased after 2 years (0.753 ± 0.145 vs 0.864 ± 0.141, *p* = 0.04), while FN and TBS showed non-significant improvement. Teriparatide was well-tolerated, with mild and transient hypercalcemia and hypophosphatemia.

**Conclusion:**

Teriparatide significantly improved BMD at the LS and TH in KTRs with osteoporosis and low bone turnover, showing a favorable safety profile.

**Supplementary Information:**

The online version contains supplementary material available at 10.1007/s11255-025-04383-8.

## Introduction

Osteoporosis is common in patients with chronic kidney disease (CKD) and the risk of fractures, including major fractures, is higher than in the general population. The incidence of fractures in patients with CKD stages 3–4, as well as those undergoing dialysis therapy or kidney transplantation, is 2–100 times higher than in age- and sex-matched individuals without CKD [1–3]. Patients with CKD who experience bone fractures have a poor quality of life and face high risks of morbidity and mortality.

Kidney transplantation is the treatment of choice for end-stage kidney disease (ESKD), since it reverses many complications of CKD and improves recipients’ quality of life and prolongs survival [4, 5]. However, bone and mineral disorders are still a burden in kidney transplant recipients (KTRs) due to preexisting alterations of bone and mineral metabolism arose during CKD progression and dialysis period, such as hyperparathyroidism and low bone turnover [6]. In addition to these alterations, bone damage is caused by immunosuppressive drugs, such as corticosteroids and calcineurin inhibitors, and modulating factors not necessarily related to kidney transplantation (e.g., gender, gonadal function, diabetes, and acid–base balance); this often results in a reduced bone mineral density (BMD) and an increased risk of fracture [7–10]. Many studies agree that the overall fracture risk after kidney transplantation is higher than in healthy individuals [11, 12]. Thus, fracture risk seems to be higher during the first 3 years after transplantation compared with patients receiving dialysis [13, 14].

The bone histomorphometric pattern in patients with ESKD, and by extension in kidney transplant candidates, has substantially changed over the past 2 decades with low bone turnover nowadays being the most prevalent condition [15–17].

Typically, after kidney transplantation, bone turnover decreases within the first year, and this reduction is associated with an increase in bone mass, unlike in patients where turnover increases [18–20]. However, in a significant portion of patients, this reduction is disproportionate, leading to an abnormal suppression of bone remodeling processes and resulting in bone loss. Many studies have found out that low bone turnover, low bone density, and generalized or focal osteomalacia are frequent histologic features in transplanted patients [9, 21, 22]. Although data are lacking to support how low bone turnover may increase the risk of cardiovascular or all-cause mortality, as well as the risk of additional fractures in KTR with osteoporosis or fracture, treating patients with low bone turnover may be challenging as anti-resorptive agents are unlikely to provide a benefit, whereas osteoanabolic agents could prove to be more effective [23].

Bone biopsy remains the gold standard for diagnosing bone disease in CKD, as it provides insights into the pathophysiological mechanisms underlying bone loss. However, it is currently available only at selected centers, limiting its routine use. A 2017 update of the Kidney Disease: Improving Global Outcomes (KDIGO) guideline states that the inability to perform a bone biopsy does not justify therapeutic nihilism when managing patients at high risk of fracture [24]. Therefore, the limited availability of routine bone biopsies has led to reliance on alternative diagnostic methods.

While dual-energy X-ray absorptiometry (DEXA) has become widely used for assessing areal bone mineral density (aBMD), the evaluation of bone turnover relies on the use of serum and urinary biomarkers, universally known as bone turnover markers (BTMs). Several studies have now demonstrated that these markers correlate with the histological pattern of bone disease determined by iliac crest biopsies, both in patients with CKD and in KTRs [25–27].

Teriparatide [recombinant human PTH-(1–34)] is a fragment of the natural human parathyroid hormone (PTH) consisting of the first 34 amino acids counting from the N-terminus end of the natural PTH. Intermittent therapy with teriparatide increases the number of osteoblasts and subsequent bone formation, an effect that is mediated by the decrease in osteoblast apoptosis and an increased activation of osteoblasts and preosteoblast. In non-CKD population, teriparatide increases bone turnover markers (BTMs) and BMD values at the lumbar spine (LS), femoral neck (FN) and total hip (TH) and for this reason, it is used in the treatment of osteoporosis with a high risk of fracture [28].

There are few data in the literature on the use of teriparatide in CKD, and even less in KTRs. We present a retrospective study evaluating the efficacy and safety of this drug in KTRs with osteoporosis and a history of fractures with low bone turnover.

## Materials and methods

### Study design

This retrospective observational study included KTR with osteoporosis complicated by fractures and low bone turnover, as defined by low levels of bone turnover markers, who were treated with teriparatide from 2019 to 2023 at the IRCCS Azienda Ospedaliero-Universitaria Policlinico Sant'Orsola by the Nephrology, Dialysis and Renal Transplant Unit and the Endocrinology and Diabetes Prevention and Care Unit.

The main inclusion criteria for the study were: KTR with stable graft function [estimated glomerular filtration rate (eGFR) above 30 mL/min/1.73 m^2^ and with no sign of rapid decline in the previous year], osteoporosis (defined as a T-score lower than − 2.5 at the L1-L4 LS, TH, or FN), a history of vertebral or non-vertebral fractures with a high fracture risk, low bone turnover at baseline, and age over 18 years. All patients provided written informed consent.

Included patients had a follow-up of at least 1 year and up to a maximum of 2 years, in accordance with the recommended treatment duration for teriparatide.

For all KTR, chronic immunosuppression was achieved with oral glucocorticoids, a calcineurin inhibitor, and antimetabolite. For induction immunosuppression, intravenous methylprednisolone was administered on the day of transplantation, followed by a subsequent tapering regimen (250 mg on day 0, 125 mg on day 1 and 2, 60 mg on day 3 and 4, 40 mg on day 5 and 6, 20 mg on day 7 and 8 and subsequent oral prednisone 25 mg with tapering to 5 mg). In addition, induction therapy included either basiliximab or thymoglobulin, based on clinical decision and the recipient’s immunological profile.

The prescribed formulation of teriparatide was Forsteo, a recombinant form of human parathyroid hormone (PTH 1–34) produced by Eli Lilly and Company (United States). The dosage followed the standard prescribing information, with 20 µg administered subcutaneously once daily.

The criterion for low bone turnover at baseline was defined based on thresholds established from Salam et al., where serum BTMs were correlated with histomorphometric data obtained from bone biopsies in CKD patients [26]. Specifically, the cut-off values for better low bone turnover discrimination were as follows: tartrate-resistant acid phosphatase 5b (TRAP5b) < 4.6 U/L, intact procollagen type I N-terminal propeptide (PINP1) < 57 ng/mL, and bone alkaline phosphatase (BAP) < 21 µg/L.

Before therapy initiation, patients were required to meet these criteria for at least two serial measurements of BTMs to ensure consistent assessment of the trend of BTMs. Prior to measuring the BTMs and determining low bone turnover, it was verified that patients were not undergoing treatment with vitamin D receptor activators (VDRAs) or calcimimetics, which could lead to over suppression of PTH and, consequently, bone turnover. Prior kidney transplantation, during hemodialysis period, no patient was treated with calcimimetics for hyperparathyroidism. Medication included only VDRA and both calcium and non-calcium-based phosphate binders.

The aim of this study is to evaluate the efficacy of teriparatide in KTR by assessing gains in aBMD at LS, TH, and FN. In addition, the study aims to assess the safety of teriparatide in this population by comparing the incidence of adverse effects (AEs) with those observed in the general population.

The study was conducted in agreement with the principles outlined in the Declaration of Helsinki and received approval from the Ethical Committee of the IRCCS Policlinico Sant’Orsola Hospital-University of Bologna (study ID: 586/2023/Oss/AOUBo). Written informed consent was obtained from all participants.

### Data collection

All data, including patient demographic characteristics, medical history, therapeutic regimens, and instrumental and laboratory assessments, were retrospectively collected from the patients’ electronic medical records.

The aBMD at LS, TH, and FN was measured using dual-energy X-ray absorptiometry (DEXA) with the GE-Lunar iDXA system (GE Healthcare, Chicago, IL, USA). The precision of the system was evaluated, with coefficients of variation (%CV) of 1.2% for LS, 1.5% for TH, and 1.8% for FN, as reported by the manufacturer and confirmed by regular phantom quality assurance tests. Daily quality assurance procedures were performed using a standard phantom, and calibration was conducted according to the manufacturer’s specifications.

The GE-Lunar iDXA system includes integrated TBS iNsight software (Medimaps Group, Geneva, Switzerland) for the evaluation of TBS, which provides an analysis of trabecular bone microarchitecture, complementing the aBMD measurements by assessing bone quality.

Data on aBMD and TBS were collected at baseline, after 1 year of teriparatide therapy, and, for patients who completed the 2-year treatment period, at 2 years.

Laboratory data included kidney function parameters such as serum creatinine and eGFR, as well as levels of calcium (Ca), phosphorus (P), albumin (for calcium adjustment), intact PTH, and 25-hydroxyvitamin D (25-OH D). BTMs assessment included serum biomarkers such as TRAP5b, PINP, BAP, and C-terminal telopeptide (CTX).

The PTH and BTMs in this study were measured using validated assays with well-defined reference ranges. PTH was assessed using the Access Intact PTH assay (Beckman Coulter, Inc.), a second-generation assay, with a reference range of 12.0–88.0 pg/mL. BAP was measured using the Access OSTASE assay (Beckman Coulter, Inc.), with a reference range of 5.7–33.0 μg/L. TRACP5b was quantified using the BoneTRAP^®^ iSYS-IDS assay, with normal values between 1.4–6.1 U/L. PINP was determined using the Intact PINP iSYS-IDS assay, with a reference range of 27.7–127.6 ng/mL. Finally, CTX was measured using the IDS-iSYS CTX-I (crossLaps) assay, with a reference range of 0.115–0.748 ng/mL (Supplementary Table 1).

All laboratory data and BTMs were measured at baseline and every 6 months throughout the study.

### Study outcomes

The primary outcome of this study was to evaluate the efficacy of teriparatide in KTR with osteoporosis and low bone turnover. Specifically, the study aimed to assess the increase in aBMD at three key skeletal sites—LS, TH, and FN—after 1 and 2 years of treatment with teriparatide. In addition, the study would explore changes in TBS at both 1 and 2 years to provide complementary information on bone microarchitecture.

The secondary outcome was to monitor the evolution of BTMs over time, investigating whether the expected increase in BTMs, typically observed with teriparatide therapy, also occurs in KTR. In particular, the study seek to determine whether the anabolic window, characterized by an early increase in both bone resorption and formation markers, even if more pronounced for bone formation markers, is similar to that seen in the non-CKD population treated with teriparatide.

The third outcome was to evaluate the safety profile of teriparatide in this specific population by monitoring the incidence and type of adverse events throughout the treatment period. The study would compare the safety data from KTR with those from the general population, focusing on the occurrence of hypercalcemia, hypotension, skin rash, and other known side effects associated with teriparatide.

### Statistical methods

Continuous variables were expressed as mean ± standard deviation (SD) or median and interquartile range [IQR], as appropriate. Categorical variables were presented as frequencies and percentages.

The normality of data distribution was assessed using the Shapiro–Wilk test. For comparisons between two groups, the unpaired Student’s *t* test was used for normally distributed variables, while the Mann–Whitney *U* test or Kruskal–Wallis test was applied for non-normally distributed variables, as appropriate. Fisher’s exact test was used to compare proportions between categorical variables.

A *p* value < 0.05 was considered statistically significant.

All statistical analyses were performed using R software (version 4.3.1).

## Results

### Baseline characteristics

A total of 18 patients were included in the study cohort. Baseline characteristics are summarized in Table 1. The mean age of the cohort was 64.4 (± 8.2) years. Of the patients, 55.5% were male (*n* = 10) and 44.5% were female (*n* = 8). Most of the cohort was Caucasian (88.8%, *n* = 16), with 11.2% being Asian (*n* = 2). The mean BMI was 24.46 (± 2.36) kg/m^2^. A history of diabetes was present in 32.3% of the cohort (*n* = 4).

**Table 1:** Baseline characteristics of patients prior teriparatide initiation

Characteristic	*N* = 18
Age, years. (± SD)	64.4 (± 8.2)
Sex, *n* (%)	
Male	10 (55.5)
Female	8 (44.5)
Ethnicity, n (%)	
Caucasian	16 (88.8)
Asian	2 (11.2)
BMI, kg/m^2^ (± SD)	24.46 (± 2.36)
Diabetes, *n* (%)	4 (32.3)
Nephropathy, *n* (%)	
Vascular	10 (55.6)
ADPKD	4 (22.2)
Glomerulonephritis	2 (11.1)
Other	2 (11.1)
Dialysis vintage, years [IQR]	5.6 [4.0–7.0]
Dialysis type, *n* (%)	
Hemodialysis	12 (66.7)
Peritoneal dialysis	4 (22.2)
Pre-emptive	2 (11.1)
Tx type, *n* (%)	
Deceased	17 (94.4)
Living	1 (5.6)
Transplant age, years [IQR]	6.5 [4.0–9.2]
LS aBMD, mg/cm^2^ (± SD)	0.941 (± 0.152)
LS T-Score [IQR]	− 1.90 (− 3.40, − 1.50)
TH aBMD, mg/cm^2^ (± SD)	0.753 [± 0.145]
TH T-Score [IQR]	− 2.60 [− 2.90, − 1.60]
FN aBMD, mg/cm^2^ (± SD)	0.742 (± 0.111)
FN T-Score [IQR]	− 2.60 [− 3.30, − 1.90]
TBS, (± SD)	1.133 (± 0.171)
Fractures, *n* (%)	18 (100)
Fractures type, *n* (%)	
Vertebral	12 (66.7)
Femoral	4 (22.2)
Other	2 (11.1)
sCreat, mg/dL (± SD)	1.35 (± 0.47)
eGFR, mL/min/1.73 m^2^ (± SD)	54.50 (± 17.56)
Ca, mg/dL (± SD)	9.32 (± 0.31)
P, mg /dL (± SD)	3.50 (± 0.61)
PTH, pg/mL (± SD)	41.50 (± 18.85)
25-OH D, ng/mL (± SD)	37.84 (± 6.45)
PINP1, ng/mL (± SD)	43.81 (± 14.80)
BAP, μg/L (± SD)	16.58 (± 8.16)
TRAP5b, U/L (± SD)	2.59 (± 0.89)
CTX, ng/mL [IQR]	0.28 [0.11–0.41]

BMI, body mass index; ADPKD, autosomal dominant polycystic kidney disease; LS, lumbar spine; TH, total hip; FN, femoral neck; TBS, trabecular bone score; sCreat, serum creatinine; eGFR, estimated glomerular filtration rate; PTH, parathyroid hormone; 25-OH D, 25-hydroxyvitamin D; PINP1, procollagen type I N-terminal propeptide; BAP, bone alkaline phosphatase; TRAP5b, tartrate-resistant acid phosphatase 5b; CTX, C-terminal telopeptide of type I collagen; SD, standard deviation; IQR, interquartile range

The median dialysis vintage was 5.6 years [IQR 4.0–7.0], with most patients on hemodialysis (66.7%, *n* = 12), followed by peritoneal dialysis (22.2%, *n* = 4), and 11.1% (*n* = 2) receiving pre-emptive transplants. Deceased-donor transplants were predominant, performed in 94.4% of cases (*n* = 17), with 5.6% receiving living donor transplants (*n* = 1). The median time since transplant was 6.5 years [IQR 4.0–9.2].

Regarding kidney function, the mean serum creatinine was 1.35 (± 0.47) mg/dL, with a mean eGFR of 54.50 (± 17.56) mL/min/1.73m^2^. The mean serum calcium was 9.32 (± 0.31) mg/dL, and the mean phosphorus level was 3.50 (± 0.61) mg/dL. PTH levels averaged 41.50 (± 18.85) pg/mL and 25-OH D 37.84 (± 6.45) ng/mL.

At baseline, the mean aBMD values were 0.94 (± 0.15) g/cm^2^ for the LS, 0.75 (± 0.14) g/cm^2^ for the TH, and 0.74 (± 0.11) g/cm^2^ for the FN. The median T-scores were − 1.90 [− 3.40, − 1.50] for the LS, − 2.60 [− 2.90, − 1.60] for the TH, and − 2.60 [− 3.30, − 1.90] for the FN. The mean TBS value was 1.13 (± 0.17).

All patients had experienced fractures (100%), with vertebral fractures in 66.7% (*n* = 12), femoral fractures in 22.2% (*n* = 4), and other types of fractures in 11.1% (*n* = 2).

BTMs showed the following mean values: PINP1 43.81 (± 14.80) ng/mL, BAP 16.58 (± 8.16) μg/L, TRAP5b 2.59 (± 0.89) U/L, and CTX with a median value of 0.28 [IQR 0.11–0.41] ng/mL.

All 18 patients underwent a 1-year follow-up after starting teriparatide, of whom 9 completed a 2-year follow-up.

### Teriparatide efficacy

**Fig. 1:**
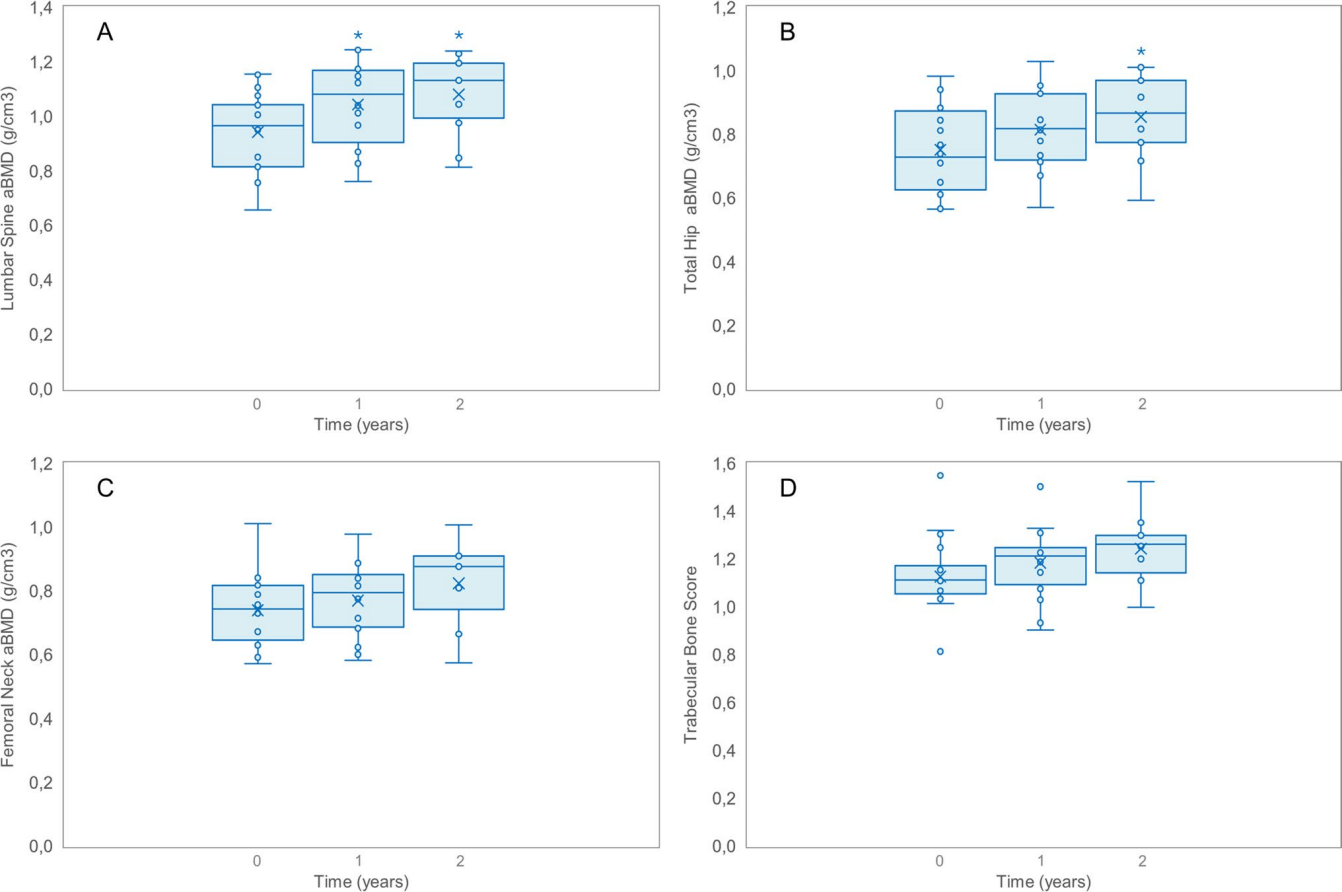
Box plots of aBMD values at lumbar spine (**A**), total hip (**B**), and femoral neck (**C**), and of TBS (**D**) at baseline, 1 year, and 2 years of teriparatide therapy. * Indicates a *p* value < 0.05 compared to baseline

For the LS, a significant increase in aBMD was observed after 1 year of teriparatide therapy compared to baseline (0.941 ± 0.152 vs 1.043 ± 0.165, *p* = 0.04) (Fig. 1). After 2 years of therapy, an additional increase in aBMD was noted (1.074 ± 0.154), although this was not statistically significant compared to the 1-year value (*p* = 0.6). However, the aBMD gain after 2 years remained statistically significant compared to baseline (0.941 ± 0.152 vs 1.074 ± 0.154, *p* = 0.03). Overall, the percentage gain in LS aBMD was 10.8% in 1 year and 14.1% at end of treatment.

For the TH, an increase in aBMD was observed after 1 year of therapy, although it was not statistically significant (0.753 ± 0.145 vs 0.832 ± 0.155, *p* = 0.1). After 2 years, a further increase in aBMD was noted (0.86 ± 0.14), which was not statistically significant compared to the 1-year value (0.832 ± 0.155 vs 0.867 ± 0.144, *p* = 0.6), but the overall gain was statistically significant compared to baseline (0.753 ± 0.145 vs 0.864 ± 0.141, *p* = 0.04). Overall, the percentage increase in TH aBMD was 10.4% in 1 year and 14.7% in 2 years.

For the FN and TBS, a trend toward improvement was observed over the 2 years of therapy. However, the overall gain for both parameters was not statistically significant compared to baseline.

A significant increase in all BTMs was observed starting from 6 months and remained significant throughout the 2-year period (Fig. 1, Supplementary Table 2).

**Fig. 2:**
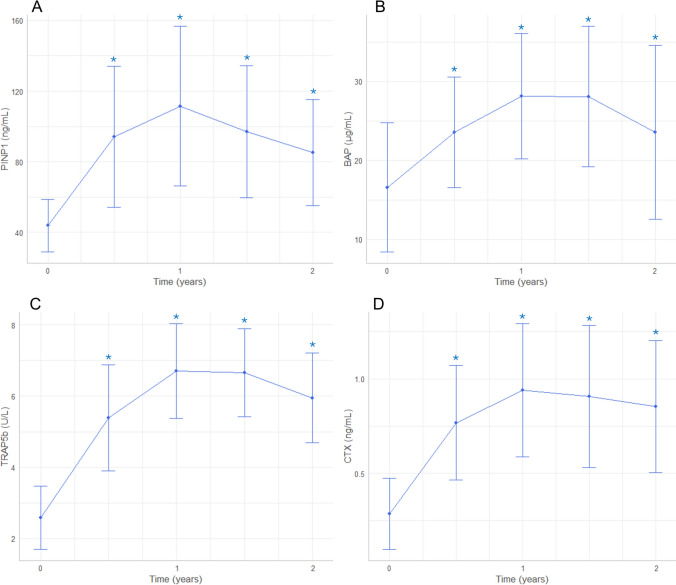
Trends of the main BTMs, PINP1 (**A**), BAP (**B**), TRAP5b (**C**), CTX (**D**), over 2 years of teriparatide therapy. As shown in the graphs, the peak of turnover is reached at 1 year of therapy, followed by a subsequent decrease, especially in the bone formation markers, while the resorption markers tend to remain higher, as at the end of the anabolic window. * Indicates a *p* value < 0.05 compared to baseline

The zenith for all BTMs occurred at 1 year, with the following mean values: PINP1 111.51 ± 45.26 ng/mL (*p* vs baseline < 0.001), BAP 28.12 ± 7.96 µg/L (*p* vs baseline < 0.001), TRAP5b 6.71 ± 1.33 U/L (*p* vs baseline < 0.001), and median CTX 0.95 [0.55, 1.13] ng/mL (*p* vs baseline < 0.001). After reaching this peak at 1 year, a decline in all BTMs was observed, but this decrease was not significant at either 18 or 24 months. By the end of the 2-year follow-up, all BTM values remained significantly higher compared to baseline (Fig. 2).

eGFR, PTH, and *p* levels remained stable throughout the follow-up period, with no statistically significant changes compared to pre-treatment values (Supplementary Fig. 1, Supplementary Table 2). A significant increase in calcium levels was observed compared to baseline at both 6 months (9.32 ± 0.28 vs 9.64 ± 0.38 mg/dL, *p* = 0.008) and 1 year (9.63 ± 0.34 mg/dL, *p* = 0.007). Following this peak, a decrease in calcium levels was noted, with values at 2 years returning to baseline levels (9.32 ± 0.44 mg/dL), showing no statistically significant difference (*p* = 0.999).

### Teriparatide safety

The AEs observed in the study are summarized in Table 2. Skin rash was reported in two patients (11.1%), and hypercalcemia occurred in four patients (22.2%), all of which were mild (below 11.0 mg/dL) and asymptomatic. Hypophosphatemia was found in three patients (16.6%), also mild (above 3.0 mg/dL) and asymptomatic. New fractures were noted in only one patient (5.5%).

**Table 2:** Adverse events reported with teriparatide

AEs	*N* (%)
Transient hypotension	0 (0.0)
Musculoskeletal disorders	0 (0.0)
Headache	1 (5.5)
Fever	0 (0.0)
Palpitation	0 (0.0)
Skin rash	2 (11.1)
Nausea	0 (0.0)
Hypercalcemia	4 (22.2)
Mild (Ca < 11.0 mg/dL)	4 (100.0)
Severe (Ca > 11.0 mg/dL)	0 (0.0)
Hypophosphatemia	3 (16.6)
Mild (*p* > 3.0 mg/dL)	3 (100.0)
Severe (*p* < 3.0 mg/dL)	0 (0.0)
New fractures	1 (5.5)
Discontinuation due to AEs	0 (0.0)

AEs, adverse events; Ca, calcium; P, phosphorus

Teriparatide was well-tolerated overall, with no patients discontinuing the medication due to AEs. There were no cases of transient hypotension, fever, palpitation, headache, or nausea.

### End of treatment

At the time of the analysis, nine patients had completed a full 24-month course of teriparatide treatment. All of them were subsequently switched to another anti-fracture drug: six patients received sequential denosumab, two received zoledronic acid, and one received romosozumab.

## Discussion

### General considerations

In this single-center, retrospective observational study, we aimed to evaluate the efficacy and safety of teriparatide in KTRs affected by osteoporosis with low bone turnover, as defined by low levels of bone turnover markers, and a history of vertebral or non-vertebral fractures.

As discussed in the inclusion criteria, in our center, we treat patients with osteoporosis and previous fractures with teriparatide only when they present with a homogeneous trend of low bone turnover in the absence of therapies that might excessively suppress PTH. Our practice is in line with previous studies [26, 27]. Expert consensus recommends that in patients with CKD or KTRs, osteoanabolic treatment should be used when low bone turnover is documented, whereas in patients with high turnover, the use of anti-resorptive agents such as bisphosphonates or denosumab is preferred [29].

Although bone biopsy is the gold standard for diagnosing bone disease phenotypes and distinguishing turnover, due to its limited accessibility, the use of BTMs has become common practice and is well-documented in the literature [25–27]. At our center as well, we do not have the availability to perform routine bone biopsies, which we reserve for specific cases. Instead, we regularly assess serum BTMs along with other bone metabolism markers (PTH, Ca, P) to evaluate bone turnover, relying not on a single measurement but on trends documented through multiple assessments.

The baseline BTMs values of the included patients were consistent with the criteria for discriminating low bone turnover assessed in bone biopsies from previous studies on CKD [25, 26]. Moreover, a subsequent study by Jørgensen et al., which performed the same type of analysis—correlating serum biomarkers with bone turnover patterns in biopsies and including KTRs—confirmed BTMs values indicative of low bone turnover in line with those we observed [27]. It should be noted that, like Salam et al. and Sprague et al., we use a second-generation intact PTH assay, whereas Jorgensen et al. used a third-generation bio-intact PTH assay [25–27].

### Teriparatide efficacy on aBMD

In terms of efficacy, a significant increase in aBMD at the LS was observed after just 1 year of teriparatide therapy with a 10.8% increase. This improvement was maintained over the 2-year period without further significant increases with an overall gain of 14.1%. Regarding the TH, we observed that the improvement, which began to show a positive trend in the first year with a 10.4% increase, reached statistical significance at the completion of 2 years of therapy with a 14.7% increase. However, for the FN and TBS, although an improving trend was noted, the sample size in this study was insufficient to register a statistically significant improvement.

There are very limited data to compare with our findings on the use of teriparatide in CKD, primarily derived from secondary analyses of registration trials and small observational studies [30–33]. Furthermore, there are currently very few studies specifically focused on its use in KTRs [34].

Regarding LS, previous studies in CKD patients are consistent with our findings. Indeed, all studies have observed an improvement in aBMD at this site [30–33]. The increase in aBMD at the LS appears to be very rapid. In fact, both Sumida et al. and Yamamoto et al. reported a significant increase at this site as early as 6 months of treatment in patients with ESKD on hemodialysis [32, 33].

As for the FN, the data on CKD are conflicting. In an exploratory analysis of the Fracture Prevention Trial, patients treated with teriparatide 20 mcg/day and 40 mcg/day versus placebo were analyzed and stratified by kidney function. In 63 patients with an eGFR between 30 and 59 mL/min/1.73 m^2^, no significant increase in aBMD at the FN was observed after 12 months of therapy, although there was a trend toward improvement compared to placebo [31].

Sumida et al., in a cohort of 22 hemodialysis patients with hypoparathyroidism who received teriparatide 56 mcg once weekly for 48 weeks, did not observe significant increases in aBMD at the FN [33]. Similarly, Yamamoto et al., in a cohort of 15 hemodialysis patients with low PTH receiving 56 mcg once weekly, did not observe any improvement at the FN, which even tended to decrease, although not significantly, at 12 months compared to baseline [32]. Our data are comparable to what was observed in the study by Miller et al., where teriparatide, in patients with reduced but not severely reduced kidney function (unlike those on hemodialysis), appears to have a beneficial effect on the FN, although with an increase that is not statistically significant [31].

No other study has analyzed the effect of teriparatide on TH and even less so the TBS. Our finding that teriparatide significantly improves aBMD at the TH after 2 years of therapy is certainly interesting and aligns with the improving trend we also observed at the FN. It is well known that trabecular bone, such as that in the LS, is much more metabolically active than cortical bone, like that in the FN or TH; therefore, it could be expected that improvements at these latter skeletal sites would take more time to occur as we observed [35]. As for the TBS, we similarly observed a trend of improvement, although not statistically significant.

The only study involving the use of teriparatide in KTRs is by Ceijka et al., where teriparatide was used immediately after kidney transplantation, as soon as kidney function began to normalize (sCreat < 2 mg/dL), for the prevention of post-transplant bone mass loss [34]. The authors observed no bone mass gain at either LS or FN compared to the controls. The study presents several design differences that make direct comparison with our results impossible. First, they used teriparatide immediately after the transplant, whereas our patients had undergone transplantation several years prior and had stable kidney function. Second, although they performed bone biopsies at the time of the transplant, osteoanabolic therapy was given a priori, without discrimination of bone turnover. It is now well established that the rapid loss of bone mass post-transplant occurs in patients where turnover tends to increase, whereas BMD is preserved in those where turnover decreases [17, 20]. Therefore, it is likely that the lack of efficacy of teriparatide therapy observed in the study, in addition to the small sample size, is attributable to the inappropriate use of osteoanabolic during a particular phase, such as the immediate post-transplant period, where high turnover seems to be the main culprit in bone damage.

### Effects of teriparatide on BTMs

Regarding the response of bone turnover during teriparatide treatment, the data on the behavior of BTMs and other bone biomarkers in previous studies are inconsistent and often conflicting. Miller et al. observed a significant increase in PINP at just 3 months of therapy with teriparatide [31]. Similarly, Sumida et al. noted an early significant increase in both PINP and BAP, although these markers tended to decrease over the course of 48 weeks [33].

Interestingly, Sumida et al. reported a decrease in TRAP5b, which was even statistically significant compared to placebo at 48 weeks. Similar results were observed by Yamamoto et al., where BAP also increased early, only to decrease over the course of 12 months, returning to baseline values [32]. In addition, bone resorption markers, TRAP5b and NTX, decreased compared to controls.

We documented a significant increase in both bone formation markers—PINP1 and BAP—and bone resorption markers—TRAP5b and CTX. The zenith was reached at 12 months of therapy, followed by a subsequent decrease in all biomarkers, with a more pronounced reduction in bone formation markers.

The behavior of BTMs identified in our study is consistent with the mechanism of action of teriparatide postulated in the general population, namely the ‘anabolic window’ [36]. The anabolic window refers to the early phase of treatment during which bone formation markers rise more significantly than bone resorption markers, leading to a net gain in bone density. Both formation and resorption markers increase, but the anabolic (bone-building) effect predominates. Typically, after about 2 years of therapy, the effects of bone formation and resorption converge, marking the end of this anabolic window.

While Miller et al. provided limited insight into whether the anabolic window was achieved, given that only PINP1 levels were reported, the studies by Sumida et al. and Yamamoto et al. show a pattern of bone resorption markers decreasing over time [31–33].

It is important to highlight that in the study by Miller et al., the teriparatide dosage was 20 or 40 mcg daily in a cohort of patients with moderately reduced kidney function (eGFR 30–59 mL/min/1.73 m^2^), whereas the studies by Sumida et al. and Yamamoto et al. included patients in ESKD [31–33]. Moreover, in these works, the teriparatide dosage was 56 mcg weekly. In our study, we used the guideline-recommended dosage of 20 µg daily in KTRs with mild to moderate reduced kidney function (mean eGFR 54.50 mL/min/1.73 m^2^). Currently, we do not know whether the different behavior of BTMs in various clinical settings may be influenced by the differences in drug dosage and its effectiveness across different spectrums of kidney function. Indeed, there are no studies investigating the pharmacokinetics of the drug in more advanced stages of kidney disease; therefore, our observations remain speculative.

### Teriparatide safety

As for AEs, the frequency of the main ones is not higher than that observed in the general population [37]. Mild hypercalcemia was one of the most common adverse events, as also reported in scientific literature for patients without CKD. This condition, while asymptomatic, was also non-persistent; it resolved on its own without requiring specific treatment or discontinuation of teriparatide. Hypophosphatemia, although less frequently reported in the literature, occurred in three patients and was mild and asymptomatic. Like hypercalcemia, it resolved spontaneously without intervention.

Interestingly, both Sumida et al. and Yamamoto et al. reported a high incidence of transient hypotension, reaching up to 40%, and treatment discontinuation in nearly 40–45% of patients due to AEs [32, 33]. In contrast, we did not observe any instances of transient hypotension in our study. It remains unclear whether factors such as the state of ESKD or the higher, albeit weekly, dosage of teriparatide used in those studies may have influenced this condition. None of our patients discontinued treatment due to severe AEs.

## Conclusion

In conclusion, our study documents the efficacy and safety of teriparatide in KTRs with osteoporosis and a history of fractures and low bone turnover. This study contributes to the existing knowledge on the use of the drug in CKD patients, confirming its efficacy, especially at the LS. In addition, it provides new data on its effectiveness at the TH.

The strengths of the study include a longer follow-up period compared to previous works, the expanded analysis of bone sites including the TH, the inclusion of bone quality assessment using TBS, and the availability of sequential analyses of all major BTMs.

The limitations of the study are the retrospective nature, the small sample size, and the lack of a control group with which to compare the results and analyze a potential reduction in fracture risk. Thus, bone turnover assessment was carried out with only serum BTMs without the implementation of bone biopsy.

Further research is needed to confirm these findings, particularly in larger cohorts of KTRs. In particular, it may be useful to evaluate the use of osteoanabolic agents versus anti-resorptive agents in cohorts of patients with low bone turnover to definitively determine the best therapeutic option.

## Supplementary Information

Below is the link to the electronic supplementary material.

Supplementary file1 (DOCX 212 KB)

## Data Availability

The data presented in this study are available on request from the corresponding author. The data are not publicly available due to privacy concern.

## Abbreviations

AEs: Adverse events
aBMD: Areal bone mineral density
BAP: Bone alkaline phosphatase
BMD: Bone mineral density
BTMs: Bone turnover markers
CKD: Chronic kidney disease
CTX: C-terminal telopeptide
DEXA: Dual-energy X-ray absorptiometry
eGFR: Estimated glomerular filtration rate
ESKD: End-stage kidney disease
FN: Femoral neck
IQR: Interquartile range
KTR: Kidney transplant recipient
LS: Lumbar spine
PTH: Parathyroid hormone
PINP: Intact procollagen type I N-terminal propeptide
SD: Standard deviation
TBS: Trabecular bone score
TH: Total hip
TRAP5b: Tartrate-resistant acid phosphatase 5b
VDRAs: Vitamin D receptor activators
