# Supplementary material for: Efficacy and safety of teriparatide in kidney transplant recipients with osteoporosis and low bone turnover: a real-world experience
Source: Int Urol Nephrol. 2025 Jan 27;57(6):1965–75. doi: 10.1007/s11255-025-04383-8 (PMC12049390; doi:10.1007/s11255-025-04383-8)
Supplement: Supplementary file 1 — Supplementary file1 (DOCX 212 KB) [file 11255_2025_4383_MOESM1_ESM.docx]

**Supplementary Table 1. BTMs and PTH assays**

| **Characteristic** | **Assay** | **Unit** | **NR** |
| --- | --- | --- | --- |
| **PTH** | Access Intact PTH, Beckman Coulter, Inc | *pg/mL* | 12.0 – 88.0 |
| **BAP** | Access OSTASE, Beckman Coulter, Inc | *μg/L* | 5.7 – 33.0 |
| **TRACP5b** | BoneTRAP® iSYS-IDS | *U/L* | 1.4 – 6.1 |
| **PINP1** | Intact PINP iSYS-IDS | *ng/mL* | 27.7 – 127.6 |
| **CTX** | IDS-iSYS CTX-I (crossLaps) | *ng/mL* | 0.115 - 0.748 |

PTH: Parathyroid Hormone; PINP1: Procollagen Type I N-Terminal Propeptide; BAP: Bone Alkaline Phosphatase; TRAP5b: Tartrate-Resistant Acid Phosphatase 5b; CTX: C-Terminal Telopeptide of Type I Collagen.

**Supplementary Table 2. BTMs and laboratory trends over time**

|  | **T0**  **N=18** | **T6**  **N=18** | **p**  **vs T0** | **T12**  **N=18** | **p**  **vs T0** | **p**  **vs T6** | **T18**  **N=9** | **p**  **Vs T0** | **p**  **vs T12** | **T24**  **N=9** | **p**  **vs T0** | **p**  **vs T12** |
| --- | --- | --- | --- | --- | --- | --- | --- | --- | --- | --- | --- | --- |
| **PINP1**  *ng/mL (±SD)* | 43.81  (*±*14.80) | 94.12  (*±*40.03) | <.001 | 111.51  (*±*45.26) | <.001 | .253 | 96.95  (*±*37.30) | <.001 | .395 | 85.18  (*±*30.17) | <.001 | .113 |
| **BAP**  μg/L *(±SD)* | 16.58  (*±*8.16) | 23.54  (*±*6.98) | .009 | 28.12  (*±*7.96) | <.001 | .075 | 28.08  (*±*8.91) | .002 | .991 | 23.54  (*±*11.01) | 0.045 | .227 |
| **TRAP5b**  *U/L (±SD)* | 2.59  (*±*0.89) | 5.38  (*±*1.49) | <.001 | 6.71  (*±*1.33) | <.001 | .008 | 6.66  (*±*1.24) | <.001 | .930 | 5.95  (*±*1.26) | <.001 | .155 |
| **CTX**  *ng/mL [IQR]* | 0.28  [0.11, 0.41] | 0.77  [0.58, 0.99] | <.001 | 1.01  [0.72, 1.18] | <.001 | .174 | 0.95  [0.55, 1.13] | <.001 | .823 | 0.88  [0.57, 1.08] | .001 | .533 |
| **PTH**  *pg/mL (±SD)* | 41.50  (*±*18.85) | 42.50  (*±*20.88) | .881 | 43.83  (*±*14.16) | .667 | .824 | 45.80  (*±*24.77) | .610 | .790 | 46.00  (*±*25.31) | .597 | .772 |
| **Ca**  *mg/dL (±SD)* | 9.32  (*±*0.31) | 9.64  (*±*0.38) | .008 | 9.63  (*±*0.34) | .007 | .927 | 9.50  (*±*0.42) | .207 | .369 | 9.32  (*±*0.44) | .999 | .054 |
| **P**  *mg/dL (±SD)* | 3.50  (*±*0.61) | 3.63  (*±*0.41) | .447 | 3.40  (*±*0.61) | .0625 | .185 | 3.20  (*±*0.82) | .280 | .467 | 3.32  (*±*0.51) | .460 | .744 |
| **eGFR**  *ml/min/*  *1.73m^2^ (±SD)* | 54.50  (*±*17.56) | 51.33  (*±*17.05) | .587 | 51.17  (*±*15.69) | .552 | .976 | 59.00  (*±*13.23) | .487 | .194 | 57.10  (*±*15.28) | .698 | .770 |

PINP1: Procollagen Type I N-Terminal Propeptide; BAP: Bone Alkaline Phosphatase; TRAP5b: Tartrate-Resistant Acid Phosphatase 5b; CTX: C-Terminal Telopeptide of Type I Collagen; PTH: Parathyroid Hormone; Ca: calcium; P: phosphorus; eGFR: Estimated Glomerular Filtration Rate; T0: baseline; T6: 6 months; T12: 12 months; T18: 18 months; T24: 24 months; p: p value.

**Supplementary Figure 1. Trends of Ca, P, PTH and eGFR. * indicates a p value < 0.05 compared to baseline.**

**
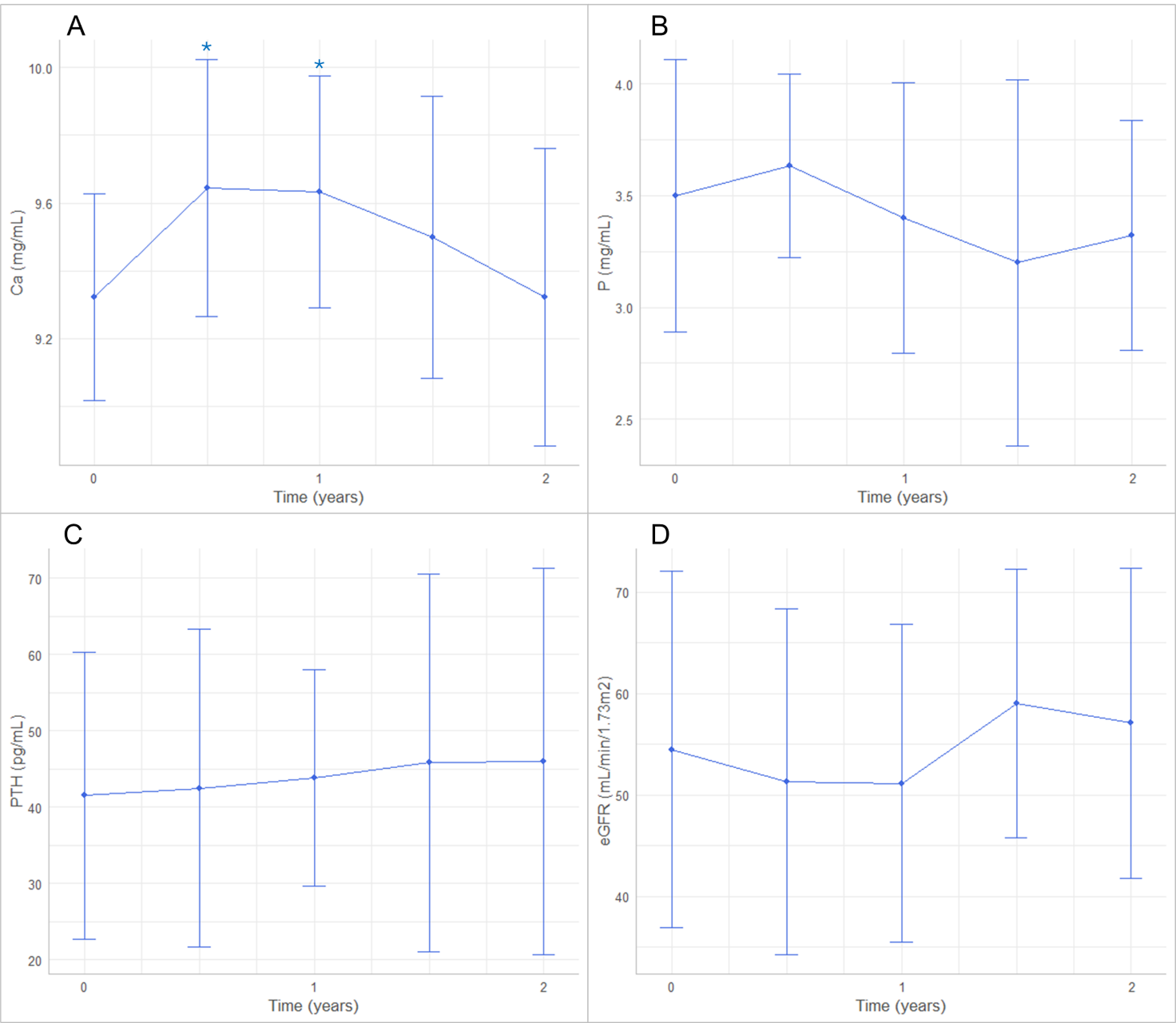
**
